# Effects of a 60-Minute Lecture About Diagnostic Errors for Medical Students: A Single-Center Interventional Study

**DOI:** 10.7759/cureus.56117

**Published:** 2024-03-13

**Authors:** Shun Yamashita, Masaki Tago, Midori Tokushima, Yoshinori Tokushima, Yuka Hirakawa, Hidetoshi Aihara, Naoko E Katsuki, Motoshi Fujiwara, Yasutomo Oda

**Affiliations:** 1 Education and Research Center for Community Medicine, Faculty of Medicine, Saga University, Saga, JPN; 2 Department of General Medicine, Saga University Hospital, Saga, JPN; 3 Saga Medical Career Support Center, Saga University Hospital, Saga, JPN

**Keywords:** patient-related factor, system-related factor, cognitive bias, medical school education, diagnostic errors

## Abstract

Introduction: The danger of diagnostic errors exists in daily medical practice, and doctors are required to avoid such errors as much as possible. Although various factors, including cognitive, system-related, and patient-related factors, are involved in the occurrence of diagnostic errors, the percentage of doctors with insufficient medical knowledge among those factors is extremely low. Therefore, lectures on diagnostic errors might also be useful for medical students without experience working as doctors. This study investigated whether a 60-minute lecture on diagnostic errors would enable Japanese medical students to consider the factors involved in diagnostic errors and how their perceptions of diagnostic errors change.

Methods and materials: This single-center interventional study was conducted in October 2022 among fourth-year medical students at the Faculty of Medicine, Saga University. A questionnaire survey was conducted before and immediately after the lecture to investigate changes in the perceptions of medical students regarding diagnostic errors. One mock case question was given on an exam the day after the lecture, and the number of responses to cognitive biases and system-related and patient-related factors involved in diagnostic errors were calculated.

Results: A total of 83 students were analyzed. After the lecture, medical students were significantly more aware of the existence of the concept of diagnostic error, the importance of learning about it, their willingness to continue learning about it, and their perception that learning about diagnostic errors improves their clinical skills. They were also significantly less likely to feel blame or shame over diagnostic errors. The mean numbers of responses per student for cognitive bias, system-related factors, and patient-related factors were 1.9, 3.4, and 0.9, respectively. The mean number of responses per student for all factors was 5.6.

Conclusion: A 60-minute lecture on diagnostic errors among medical students is beneficial because it significantly changes their perception of diagnostic errors. The results of the present study also suggest that lectures may enable Japanese medical students to consider the factors involved in diagnostic errors.

## Introduction

In the general process of medical practice, a patient visits a medical facility with health problems, and a doctor starts gathering medical information [1]. A doctor integrates and interprets medical information, makes a provisional diagnosis, explains the patient’s medical condition, and starts treatment and follow-up [1]. If the disease course of a patient differed from what was expected, the doctor repeated the process of gathering information [1]. Errors in this process can lead to “diagnostic errors,” including delayed diagnoses, misdiagnoses, and missed diagnoses [1,2]. Diagnostic errors are estimated to occur in 10-15% of U.S. patients, making medical errors, including diagnostic errors, the third leading cause of death among them [3]. In Japan, 47.5% of cases in which payment was ordered by a medical court were due to diagnostic errors, and 62.3% of such cases resulted in patient death [4]. Thus, the danger of diagnostic errors exists in daily medical practice, and doctors are required to avoid such errors as much as possible.

Cognitive (74%), system-related (65%), and patient-related factors (44%) are estimated to be involved in the occurrence of diagnostic errors [3]. Both cognitive and system-related factors are involved in 46% of diagnostic errors [3]. Insufficient medical knowledge among doctors reportedly accounts for only 4% of cognitive factors, or 3% of all factors involved in diagnostic errors [3]. Therefore, lectures on diagnostic errors could help medical students without working experience as doctors recognize the causes of diagnostic errors. In fact, it has been reported that 76% of second-year medical students in the U.S. who received a lecture for a total of 10.5 hours on patient safety overview, error reporting, system versus human approach, safety tools, and ethics/disclosure were able to observe medical errors in clinical practice after one year [5]. It has also been reported that 62% of medical students who discuss factors involved in diagnostic errors, types of errors, and the importance of prevention and reporting of errors and submit a report about diagnostic errors in a mock case could propose ways to prevent the diagnostic errors they encountered during clinical training [6]. In Japan, diagnostic errors are not included in the model core curriculum for medical education, which defines the learning goal of practical clinical capabilities required to acquire the necessary skills before graduation [7].

It is considered that increasing awareness of diagnostic errors among medical students is important because it will help prevent them and improve medical care and patient safety [8]. There have been interventional studies of virtual lectures on diagnostic errors for medical students before clinical clerkship and workshops on how to analyze diagnostic errors during clinical clerkship [6,9]. Such education has been reported to help medical students deepen their understanding of diagnostic reasoning skills and enable them to correctly analyze and report errors. However, it is especially important to create an environment in which doctors share their own error cases and do not blame other doctors who have made errors but instead examine the causes. However, to the best of our knowledge, there have been no studies of how lectures on diagnostic errors change how they are perceived by medical students and affect their motivation to learn. The present study aimed to clarify whether a lecture on factors involved in diagnostic errors and how to reflect on such errors for the 4th year medical students before clinical training changed their recognition of the concept of diagnostic errors and whether they could recognize the diagnostic error factors.

## Materials and methods

Study design and participants

This single-center interventional study included fourth-year medical students at the Faculty of Medicine, Saga University, who attended a 60-minute lecture on diagnostic errors in October 2022. Students who did not attend the lecture or agree to participate in the study were excluded from the analysis. The lecture included the following topics: definition of diagnostic errors, incidence of diagnostic errors in various settings, percentage of medical errors among causes of death, types of diseases with a high frequency of medical errors, relationship between medical trials and diagnostic errors in Japan, common diagnostic processes, factors of diagnostic errors (cognitive factors, system-related factors, patient-related factors), cognitive biases, and how to reflect on cases of diagnostic errors using the Fishbone diagram [10]. In a general internal medicine examination performed the day after the lecture, a mock case question was asked, and medical students answered questions on factors involved in diagnostic errors (Supplement 1). In addition, a survey using a questionnaire consisting of the same items was conducted before and immediately after the lecture to investigate changes in medical students’ perceptions of diagnostic errors due to the lecture.

Setting

Saga Prefecture is a rural city in Japan with a population of approximately 830,000. It is located north of Kyusyu. The present study was performed at the Faculty of Medicine, Saga University, which is the only national university with a medical school in Saga Prefecture. Medical students at the Faculty of Medicine, Saga University, take specialized courses including anatomy, microbiology, biochemistry, or pathology, as well as classroom lectures on clinical knowledge of each organ-specific disease until the fourth year's end. Education through workshops and small group discussions is not usually provided. Then, they take the objective structured clinical examination and the computer-based testing, which evaluate their basic clinical knowledge and skills [7]. Only students who pass the exams can advance to the fifth year and perform clinical training as student doctors. This lecture was delivered to fourth-year medical students as part of their curriculum. In October 2022, when the present study was performed, there were 102 fourth-year medical students at the Faculty of Medicine, Saga University, and none of them had received a lecture on diagnostic errors before this lecture.

Data sources

The questionnaire methods were made using Google Forms (Google, Mountain View, CA, USA), and the following items were investigated (Supplement 2): age, gender, whether the participant knew the concept of diagnostic error, whether the participant could explain the significance of learning about diagnostic error, whether medical students should learn about diagnostic error, whether the participant wants to keep learning about diagnostic error, whether doctors who made diagnostic errors should be blamed, whether making diagnostic errors is embarrassing, whether learning about diagnostic errors can lead to improved clinical skills, whether the diagnosis made by a specialist is more correct than a non-specialist, and whether the diagnosis made by an experienced doctor is more accurate than a young doctor. Items other than age and gender in the questionnaire were surveyed using 5-point Likert-scale questions (1 = disagree at all or do not know at all, to 5 = agree very strongly or know very well). In an exam using a mock case performed the day after the lecture, the contents of the answered cognitive bias, system-related factors, patient-related factors, and the number of responses were surveyed.

Data analysis

Continuous variables are expressed as mean and standard deviation and were compared using the t-test. Categorical variables are expressed as percentages and compared using the χ2 test. Missing values were excluded from the analysis. Changes in the perception of diagnostic errors before and after the lectures were evaluated using a paired-sample t-test. Relationships between the perception of diagnostic error after the lecture and the number of responses of cognitive bias, responses of system-related and patient-related factors, and total responses in the exam were evaluated using Spearman’s r value. Missing values were excluded from the analysis. The statistical significance was set at p<0.05. SPSS Statistics version 25 (IBM Corp. Released 2017. IBM SPSS Statistics for Windows, Version 25.0. Armonk, NY: IBM Corp.) was used for statistical analysis.

Ethical consideration

This study was approved by the Ethics Committee of the Faculty of Medicine, Saga University (approval no. R5-12). The agreement to participate in the present study was confirmed at the beginning of the questionnaire before the lecture, and only students who agreed to participate were analyzed. In addition, the present study was performed in accordance with the 1975 Declaration of Helsinki and the rule on medical ethics of the Faculty of Medicine.

## Results

Enrollment of participants

**Figure 1:**
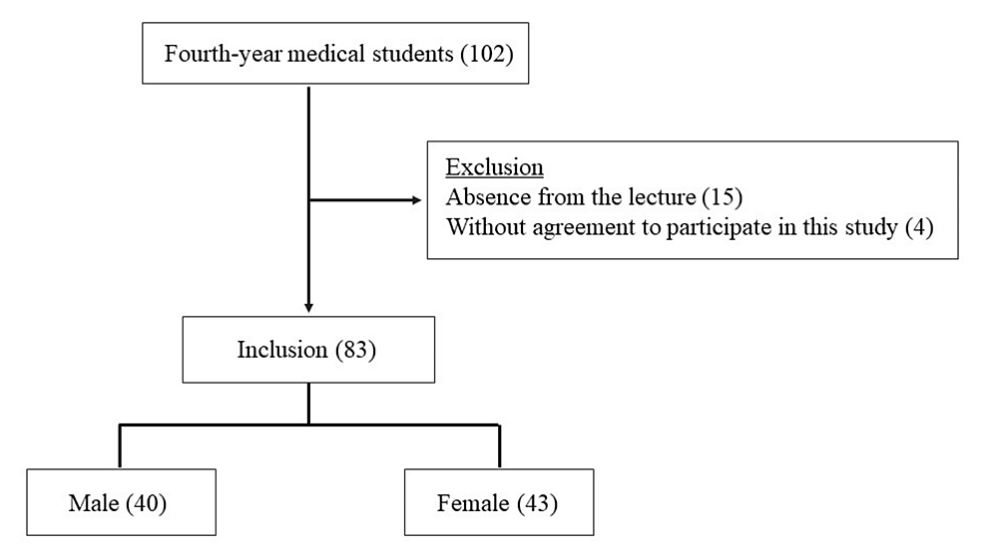
Inclusion criteria

In October 2022, when the present study was conducted, there were 102 fourth-year students in the Faculty of Medicine, Saga University. Fifteen students who were absent from the lecture and four students who refused to participate in the present study were excluded from the analysis. Finally, 83 students were enrolled in this study (Figure 1). The median age of the respondents was 22.2 ± 1.1 years, including 40 (48%) males and 43 (52%) females.

Cognitive biases, system-related factors, and patient-related factors which were responded to in the exam on the day after the lecture

The cognitive biases, system-related factors, and patient-related factors that responded to the examination are shown in Table 1. The mean number of responses for cognitive bias was 1.9 ± 1.2, most of which was the early closure of diagnosis (73%), followed by confirmation bias (53%), and representativeness bias (25%). The mean number of responses for system-related factors was 3.4 ± 0.8, most of which were from consultants having difficult communication (99%), followed by physician fatigue (95%), and busy outpatient clinics (78%). The mean number of responses for patient-related factors was 0.9 ± 0.3, including “a patient frequently visiting the emergency room with the same symptoms” (39%). The mean number of all responses for cognitive bias and system-related and patient-related factors was 5.6 ± 1.5.

**Table 1:** Cognitive biases, system-related factors, and patient-related factors as answered in the exam on the following day after the lecture Categorical data are expressed as n (%) and continuous variables are expressed as the mean ± standard deviation

	N=83
Age	22.1 ± 1.1
Cognitive biases	1.9 ± 1.2
Early closure of diagnosis	61 (73)
Confirmation bias	44 (53)
Representativeness bias	21 (25)
Anchoring bias	12 (14)
Availability bias	12 (14)
Unpacking principle error	6 (7)
Overconfidence bias	2 (2)
System-related factors	3.4 ± 0.8
Consultation being difficult communication	82 (99)
Fatigue of the doctor	79 (95)
Busy outpatient clinics	65 (78)
Post graduated year of the doctor	52 (63)
Patient-related factor	0.9 ± 0.3
A patient frequently visiting the emergency room with the same symptoms	32 (39)
A total of cognitive, system-related, and patient-related factors	5.6 ± 1.5

Changes in the recognition of diagnostic errors of medical students before and after the lecture

Changes in the recognition of diagnostic errors by medical students before and after the lecture are shown in Table 2. Medical students after the lecture were more aware of the existence of diagnostic errors (1.9 ± 1.0 vs. 4.5 ± 0.5, p<0.001) and more confident in explaining the significance of learning them (2.6 ± 1.3 vs. 4.6 ± 0.6, p<0.001) than before the lecture. In addition, they were more aware of the importance of learning about diagnostic errors during medical school (4.2 ± 0.8 vs. 4.9 ± 0.3, p<0.001), were more motivated to continue learning them in the future (4.2 ± 0.8 vs. 4.8 ± 0.5, p<0.001), and identified that learning about diagnostic errors would improve their clinical skills (4.4 ± 0.7 vs. 4.8 ± 0.4, p<0.001) than before the lecture. Recognitions that the diagnosis made by a specialist was more correct than a non-specialist (3.5 ± 0.8 vs. 2.2 ± 1.0, p<0.001) and that the diagnosis made by an experienced doctor was more correct than a younger doctor (3.2 ± 1.0 vs. 2.1 ± 0.9, p<0.001) decreased. Recognitions that a doctor who made diagnostic errors should be blamed (2.5 ± 0.8 vs. 1.9 ± 1.0, p<0.001) and that making diagnostic errors is embarrassing (2.8 ± 1.0 vs. 2.0 ± 1.0, p<0.001) also decreased.

**Table 2:** Changes in the recognition of diagnostic errors by medical students before and after a 60-minute lecture about diagnostic errors 5-point Likert-scale questions (1 = disagree at all or do not know at all to 5 = agree very strongly or know very well) were used for this questionnaire. Continuous variables are expressed as the mean ± standard deviation

	Pre	Post	p-value
I am familiar with diagnostic errors	1.9 ± 1.0	4.5 ± 0.5	<0.001
I can explain the significance of learning diagnostic errors	2.6 ± 1.3	4.6 ± 0.6	<0.001
Medical students should learn about diagnostic errors	4.2 ± 0.8	4.9 ± 0.3	<0.001
I would like to keep studying diagnostic errors in the future	4.2 ± 0.8	4.8 ± 0.5	<0.001
Doctors making a diagnostic error should be blamed	2.5 ± 0.8	1.9 ± 1.0	<0.001
Making a diagnostic error is embarrassing	2.8 ± 1.0	2.0 ± 1.0	<0.001
Learning about diagnostic errors would improve clinical skills	4.4 ± 0.7	4.8 ± 0.4	<0.001
A diagnosis made by a specialist is more correct than a non-specialist	3.5 ± 0.8	2.2 ± 1.0	<0.001
A diagnosis made by an experienced doctor is more correct than a young doctor	3.2 ± 1.0	2.1 ± 0.9	<0.001

Relationship between recognition of diagnostic errors after the lecture and the number of responses to factors of the diagnostic error in the exam

The relationship between the recognition of diagnostic errors by medical students after the lecture and the number of responses to cognitive bias, system-related factors, patient-related factors, and the total number of responses on the exam is shown in Table 3. The number of responses to cognitive bias was negatively correlated with the recognition that making a diagnostic error is embarrassing (r = -0.242, p<0.05). The sum of the responses for the system-related and patient-related factors was positively correlated with the recognition that medical students should learn about diagnostic errors (r = 0.235, p<0.05). The total number of responses for cognitive bias, system-related, and patient-related factors was positively correlated with the willingness to continue learning about diagnostic errors in the future (r = 0.240, p<0.05).

**Table 3:** Correlation between the recognition of diagnostic errors by medical students after the 60-minute lecture and the number of responses to cognitive bias, system-related factors, and total factors, including patient-related factors *p<0.05

N	Number of responses		
	Cognitive bias	System-related factors	Total
I am familiar with diagnostic errors	0.001	0.122	0.067
I can explain the significance of learning diagnostic errors	-0.131	-0.007	-0.104
Medical students should learn about diagnostic errors	-0.095	0.235*	0.064
I would like to keep studying diagnostic errors in the future	0.166	0.197	0.240*
Doctors making a diagnostic error should be blamed	-0.122	-0.076	-0.129
Making a diagnostic error is embarrassing	-0.242*	-0.016	-0.197
Learning about diagnostic errors would improve clinical skills	-0.064	0.040	-0.065
A diagnosis made by a specialist is more correct than a non-specialist	-0.154	-0.046	-0.152
A diagnosis made by an experienced doctor is more correct than a young doctor	-0.161	-0.044	-0.139

## Discussion

Diagnostic errors occur for 3.9% of primary care patients in Japan [11], which can directly lead to serious outcomes such as patient death [4]. Therefore, physicians must avoid as many diagnostic errors as possible. However, the content of diagnostic errors, which defines educational content for medical students, is not included in the model core curriculum for medical education in Japan, which defines education content for medical students [7]. Therefore, most doctors may gradually become aware of the importance of diagnostic errors after they have worked in clinical practice. To help doctors recognize the dangers of diagnostic errors similar to those in clinical practice, it is important to educate medical students about diagnostic errors [5,8]. In the U.S., there have been interventional studies of virtual lectures on diagnostic error and clinical reasoning for medical students before clinical clerkship and workshops during clinical clerkship to discuss errors observed in clinical practice and to prepare mock reports [6,9]. Such education has reportedly helped medical students deepen their understanding of diagnostic reasoning skills and enabled them to correctly analyze and report errors. However, it has remained unclear whether lectures on diagnostic errors change the recognition of medical students. This study revealed that a lecture explaining the causes of diagnostic errors and how to reflect on them may change medical students’ recognition of diagnostic errors and enable them to consider the factors involved in diagnostic errors.

Cognitive (74%), system-related (65%), and patient-related factors (44%) are representative causes of diagnostic errors [3]. In general, a total of 5.9 cognitive and system-related factors have been detected in diagnostic error cases [3]. In addition, cognitive biases are involved in more than 70% of diagnostic error cases, and the average number of cognitive biases detected in a diagnostic error case is 3.08 [3,10,12]. In the mock case questions in our study, the mean number of cognitive biases and system-related factors answered by medical students at a regional Japanese university were 1.9 and 3.4, respectively, and the mean total number of answers was 5.6. Although the number of cognitive biases answered by medical students was lower than the number of those involved in a diagnostic error case in an actual clinical setting [3,12,13], the results of this study suggest that a 60-minute lecture on diagnostic errors may enable medical students at a rural Japanese university with no clinical experience to consider factors involved in a diagnostic error case.

The lack of medical knowledge among doctors as a cause of diagnostic errors reportedly accounts for only 4% of all cognitive factors [3]. Therefore, it is not appropriate for a doctor who makes a diagnostic error not to share the diagnostic error case with others out of embarrassment or for other doctors to blame the doctor [14]. It is important to improve patient safety by recognizing the causes of diagnostic errors, improving the system to resolve the issue, and preventing future diagnostic errors [15,16]. Therefore, healthcare providers, including doctors, are required to share diagnostic error cases and learn about the factors involved in diagnostic errors [17]. The lecture on diagnostic errors in the present study has helped medical students no longer believe that making diagnostic errors is embarrassing and that doctors who make such errors should be blamed. In addition, the negative correlation between the number of responses to cognitive biases in the exam and students’ perception after the lecture that making a diagnostic error is embarrassing suggests that improving their understanding of cognitive biases may further reduce their inclination that making diagnostic errors is embarrassing. Furthermore, they believed that diagnoses made by specialists or experienced doctors were not always more accurate than those made by non-specialists and younger doctors. In fact, it has been reported that there is a positive correlation between the age of a doctor and the odds ratio of diagnostic errors [18]. This suggests that it is not easy to escape cognitive biases, even when aware of their likelihood in actual clinical practice [19], and that anyone can make diagnostic errors. The results of the present study suggest that medical students may have felt these things through the lecture on diagnostic errors. Thus, we believe that a lecture on diagnostic errors for medical students may be beneficial because it may decrease embarrassment for making diagnostic errors and blame the doctor who made them, both of which are important for improving patient safety.

The present study has several limitations. First, this was a single-center cross-sectional study conducted at a rural Japanese national university. Second, the level of understanding of cognitive biases, system-related factors, and patient-related factors was measured on the day after the lecture in the present study. Therefore, it was not possible to measure their level of understanding of these factors with the same mock case question before the lecture. Third, medical students’ understanding of diagnostic errors was not assessed in the present study. Therefore, it is unclear to what extent they understood the definition of a diagnostic error and were able to explain its significance. In addition, regarding the relationship between the recognition of diagnostic errors after the lecture and the number of responses to cognitive biases, system-related factors, and patient-related factors, the cause and effect may be reversed depending on the interpretation. Fourth, this study did not examine why the 60-minute lecture about diagnostic errors changed the perception of diagnostic errors in medical students. Fifth, we evaluated the change in medical students’ perception that making diagnostic errors is embarrassing by only using specific elements of the lecture. Therefore, different survey items may have produced different results. Finally, the questionnaire survey was administered just before and immediately after the start of the lecture, and it is unclear whether the medical students in the present study continued to learn about diagnostic errors after the lecture.

## Conclusions

A 60-minute lecture on diagnostic errors could encourage medical students before clinical clerkship to consider the cognitive biases, system-related factors, and patient-related factors involved in diagnostic errors. Such lectures could also reduce the perception that making diagnostic errors is embarrassing or that doctors who make diagnostic errors should be blamed. In particular, gaining a better understanding of cognitive biases may further reduce the embarrassment associated with making diagnostic errors.

## Appendices

Supplement 1: a mock case question the day after the lecture

Read the following medical history and describe the cognitive biases and system and patient factors that may have contributed to this diagnostic error, along with the reasons.

Medical History

On holiday, a 58-year-old man visited the emergency department with paroxysmal headaches three hours before admission. The headache gradually worsened, and he vomited immediately before the first visit to the hospital. Therefore, the patient was admitted to the emergency department without an ambulance. His level of consciousness was alert, and he had no fever. However, his blood pressure increased to 180/105 mmHg. On that day, a young doctor in his third postgraduate year, who had worked until late last night, was responsible for the diagnosis and treatment at the emergency room. In addition, when the patient visited the hospital, the outpatient clinic was filled with fever patients. Although the doctor wanted to ask the radiologist to perform a cranial computed tomography (CT) examination, he hesitated because he had previously been rebuked by the radiologist on duty on that day. A physical examination revealed no paralysis or other neurological symptoms, and the doctor determined that the headache was due to the same cause as usual. The patient was discharged without a cranial CT examination. After several hours, his headache intensified to a level that he had never previously felt. He visited the same hospital again, and cranial CT revealed a subarachnoid hemorrhage.

Supplement 2: list of questionnaire items

Five-point Likert-type scale questions (1 = disagree at all or do not know at all to 5 = agree very strongly or know very well)

・I am familiar with diagnostic errors.

・I can explain the significance of learning diagnostic errors.

・Medical students should learn about diagnostic errors.

・I would like to keep studying diagnostic errors in the future.

・Doctors making a diagnostic error should be blamed.

・Making a diagnostic error is embarrassing.

・Learning about diagnostic errors would improve clinical skills.

・A diagnosis made by a specialist is more correct than a non-specialist.

・A diagnosis made by an experienced doctor is more correct than a young doctor.
